# Polymorphism in the *GATM* Locus Associated with Dialysis-Independent Chronic Kidney Disease but Not Dialysis-Dependent Kidney Failure

**DOI:** 10.3390/genes12060834

**Published:** 2021-05-28

**Authors:** Špela Šalamon, Sebastjan Bevc, Robert Ekart, Radovan Hojs, Uroš Potočnik

**Affiliations:** 1 Center for Human Molecular Genetics and Pharmacogenomics, Faculty of Medicine, University of Maribor, Taborska ul. 8, 2000 Maribor, Slovenia; spela.salamon@kages.at; 2 Department of Nephrology, Clinic for Internal Medicine, University Medical Centre Maribor, Ljubljanska Ulica 5, 2000 Maribor, Slovenia; sebastjan.bevc@ukc-mb.si (S.B.); radovan.hojs@ukc-mb.si (R.H.); 3 Department of Internal Medicine and Department of Pharmacology, Faculty of Medicine, University of Maribor, Taborska Ulica 8, 2000 Maribor, Slovenia; robert.ekart2@guest.arnes.si; 4 Department of Dialysis, Clinic for Internal Medicine, University Medical Centre Maribor, Ljubljanska Ulica 5, 2000 Maribor, Slovenia; 5 Laboratory for Biochemistry, Molecular Biology and Genomics, Faculty for Chemistry and Chemical Engineering, University of Maribor, Smetanova ul. 17, 2000 Maribor, Slovenia

**Keywords:** chronic kidney disease (CKD), kidney failure, rs2453533, rs4293393, *GATM*, UMOD, SNP, eGFR

## Abstract

The ten most statistically significant estimated glomerular filtration rate (eGFRcrea)-associated loci from genome-wide association studies (GWAs) are tested for associations with chronic kidney disease (CKD) in 208 patients, including dialysis-independent CKD and dialysis-dependent end-stage renal disease (kidney failure). The allele A of intergenic SNP rs2453533 (near *GATM*) is more frequent in dialysis-independent CKD patients (*n* = 135, adjusted *p* = 0.020) but not dialysis-dependent kidney failure patients (*n* = 73) compared to healthy controls (*n* = 309). The allele C of intronic SNP rs4293393 (*UMOD*) is more frequent in healthy controls (adjusted *p* = 0.042) than in CKD patients. The Allele T of intronic SNP rs9895661 (*BCAS3*) is associated with decreased eGFRcys (adjusted *p* = 0.001) and eGFRcrea (adjusted *p* = 0.017). Our results provide further evidence of a genetic difference between dialysis-independent CKD and dialysis-dependent kidney failure, and add the *GATM* gene locus to the list of loci associated only with dialysis-independent CKD. *GATM* risk allele carriers in the dialysis-independent group may have a genetic susceptibility to higher creatinine production rather than increased serum creatinine due to kidney malfunction, and therefore, do not progress to dialysis-dependent kidney failure. When using eGFRcrea for CKD diagnosis, physicians might benefit from information about creatinine-increasing loci.

## 1. Introduction

Chronic kidney disease (CKD) is “the most neglected chronic disease” according to the World Health Organization, and is often treated as just a comorbidity of other diseases. In high-income countries, 2–3% of annual healthcare budgets are spent on 0.03% of the total population—patients whose disease progresses to end-stage renal disease/failure (dialysis-dependent kidney failure) requiring replacement therapy [1]. Current guidelines define CKD as a glomerular filtration rate (GFR) of less than 60 mL/min per 1.73 m^2^, kidney damage markers, or both, for at least three months [2]. The age- and sex-adjusted CKD prevalence in Europe varies widely, between <7% and >20% in the adult population aged 65–74 years [3]. Traditionally, CKD is tracked using estimated GFR (eGFR; usually calculated based on serum creatinine value, then also known as eGFRcrea, or less frequently based on serum cystatin C, then also known as eGFRcys [4]) values and clinical, laboratory and imaging features at a given point in time. CKD risk factors are classified into initiating and perpetuating factors with only a partial overlap. Known risk factors for CKD progression include albuminuria, hypertension, hyperglycemia, smoking, obesity and history of cardiovascular disease [2]. CKD patients often suffer from common comorbidities including arterial hypertension, diabetes mellitus and cardiovascular disease. These conditions result in a harmful internal environment, especially when they coexist [5]. Initiating factors contribute predominantly to starting the cycle of nephron loss while perpetuating factors mainly contribute to disease progression [6]. Consensus on a definition of CKD progression has not been established and reliable biomarkers for early CKD progression prediction are still lacking. Development of dialysis-dependent kidney failure, often defined by requirement for dialysis or transplantation, is the only universally recognized clinical endpoint for CKD [7]. Therefore, comparing clinically similar groups of dialysis-dependent kidney failure against CKD patients with similar characteristics who have not developed dialysis-dependent kidney failure, is an indirect way to study CKD progression. Proteinuria or albuminuria (even more relevantly measured as change in albuminuria) [8] is also a popular surrogate clinical endpoint for CKD due to its well-established association with kidney function decline. 

Genome-wide association studies (GWAs) search for associations between genetic loci and CKD-defining traits (CKD-dt) [9] including eGFRcrea measurements or CKD based on the definition of eGFRcrea under 60 mL/min per 1.73 m^2^ [10]. The latest and largest GWA meta-analysis of eGFRcrea-associated loci on over a million individuals has identified 308 eGFR variation loci that collectively explain about 7% of eGFR heritability in the general population [11]. Some of the most statistically significant CKD-dt single nucleotide polymorphisms (SNPs) from GWAs lack replications in CKD patient cohorts, especially in studies that distinguish between dialysis-independent CKD and dialysis-dependent kidney failure, or include measures of disease progression or other relevant clinical parameters. Böger et al. [12] suggested differences in genetic architecture between dialysis-dependent kidney failure and dialysis-independent CKD patients and reported a few candidate genes associated only with dialysis-independent CKD but not dialysis-dependent kidney failure. Surprisingly, so far, very few GWAs and candidate gene association studies have compared genetic risk factors separately in dialysis-dependent kidney failure and dialysis-independent CKD to verify this initial finding. To further investigate the potential difference in genetic architecture between subgroups of CKD patients, in this study, we compare the genotypes of 12 leading GWAs’ SNPs from 10 loci in cohorts of Slovenian dialysis-independent CKD and dialysis-dependent kidney failure patients and Slovenian disease-free control individuals. Additionally, we interrogate the same SNPs for associations with eGFR in dialysis-independent CKD. We aim to make the dialysis-independent and dialysis-dependent groups comparable to each other in terms of age, sex, comorbidity, systolic blood pressure, extent of edema and body mass index (BMI).

## 2. Materials and Methods

We enrolled 208 Slovenian CKD patients, including 73 dialysis-dependent kidney failure patients receiving hemodialysis and 135 dialysis-independent patients treated in the outpatient nephrology clinic of the University Medical Centre Maribor (UMCM), Slovenia. As the control group, 309 healthy Slovenian blood donors from the UMCM were available. The inclusion criteria for patients were a CKD diagnosis of any CKD stage ranging from one to five according to the KDOQI Clinical Practice Guidelines [13] for Chronic Kidney Disease (or dialysis-dependent kidney failure requiring dialysis) and that the patients must be of Slovenian origin. Patients with missing patient records were excluded. Clinical information was extracted from UMCM patient clinical records. Retrospective values for eGFRcrea, eGFRcys, albuminuria, systolic blood pressure and BMI were recorded over a period of 5 ± 3 years. eGFRcrea and eGFRcys were measured at recruitment and then within three months of regular one-year intervals. After five such measurements, all values were averaged for each patient to generate eGFR values that were normalized over time. The most common comorbidities—arterial hypertension, diabetes mellitus and cardiovascular disease—were also recorded [5]. Presence of arterial hypertension and/or diabetes mellitus was defined as a diagnosis of arterial hypertension/diabetes mellitus in the patient records wherein we included both T1D (type 1 diabetes) and T2D (type 2 diabetes) patients. Presence of cardiovascular disease was determined as any of the following in the records: diagnosis of coronary heart disease or heart failure, descriptions of cardiac events including all aspects of acute coronary syndrome, history of arrhythmias that required hospital treatment or pacemaker implantation, sonographic evidence of heart failure or unequivocal post-ischemic ECG changes. Albuminuria was measured with dipsticks on a semi-quantitative scale from zero to four, where zero is 0 g/L, one is 0.3 g/L, two is 1.0 g/L, three is 3.0 g/L and four is > 3.0 g/L of urinary albumin. Numeric parameters were averaged over each individual’s respective observation period. The mean observation period for clinical parameters was 5±3 years for each patient unless otherwise indicated. Serum creatinine was measured using the kinetic method according to the Jaffé method without deproteinization (isotope dilution mass spectrometry traceable method; Roche Diagnostics, Mannheim, Germany). This is a compensated method based on manufacturer instructions as described previously [14]. Serum cystatin C was measured using the particle-enhanced immunonephelometric method (Dade Behring, Marburg, Germany). The values were recalculated to the certified reference standard using the multiplication factor according to the manufacturer’s specifications.

We recruited the individuals in this study during routine control visits to the outpatient nephrology clinic (dialysis-independent) and dialysis center (dialysis-dependent) of the UMCM between 10.2.2015 and 19.5.2016. 

We included patients:

1. With the clinical diagnosis of CKD, regardless of the severity of kidney function impairment
2. Of local ancestral background
3. Able to understand and sign informed consent

We excluded patients:

1. With active malignant disease (patients with a history of malignant disease considered cured or in remission were included)
2. Aged below 18 years
3. With missing medical records

Participant data acquisition and processing were performed at the UMCM. Laboratory analyses were performed at the Center for Human Molecular Genetics and Pharmacogenomics, Faculty of Medicine, University of Maribor, Slovenia.

Ethics approval and consent to participate: All patients and healthy individuals agreed to participate prior to inclusion in the study with signed informed consent. The study was in accordance with The Code of Ethics of the World Medical Association (Declaration of Helsinki) and approved by the Republic of Slovenia National Medical Ethics Committee, Ministry of Health, with the reference number 110/02/15.

The characteristics of the included individuals and their clinical parameters are presented in Table 1. 

We isolated the DNA from 9 mL peripheral blood. The peripheral blood lymphocytes were collected using density cell separation media. Subsequently, DNA was isolated from lymphocytes using a monophase solution containing phenol and guanidine thiocyanate. All nucleic acid samples were quantified and diluted to equal concentrations using a BioTek Synergy2 Microplate Reader (BioTek, Winooski, VT, USA).

### 2.1. SNP Selection and Genotyping

The ten most significant eGFRcrea loci from the largest GWA meta-analysis [11] (according to the meta-analysis p-values) were chosen for replication. Where genotypes for selected SNPs were not available, the SNPs were substituted with high-linkage disequilibrium (high-LD) SNPs (calculated with LDlink software) for European populations. The SNPs with their D’ and R^2^ values are shown in (Supplementary Table S1) [15]. In the case of the *GATM* and *UMOD/PDILT* loci, we also included the following additional SNPs near our lead SNPs: a lower LD SNP rs2453533-*GATM*, because the SNP has been known as a significant CKD-related polymorphism since the historic pair of CKD GWAs by Köttgen et al. [16,17] and a suitable digestion enzyme was readily available; and a higher LD SNP rs11864909 *UMOD/PDILT* for calculation control purposes. Therefore, our analysis included 12 SNPs. The selected SNPs and their LD relation to the SNPs from the largest GWA meta-analysis [11] are presented in (Supplementary Table S1). 

Genotyping was performed on 208 patients, including 73 dialysis-dependent kidney failure and 135 dialysis-independent CKD patients. For SNPs rs2453533-*GATM*, rs4293393-*UMOD/PDILT*, rs1047891-*CPS1* and rs989566-*BCAS3*, 309 genotypes of healthy unrelated control individuals were available. For all other SNPs, 125 genotypes of healthy unrelated control individuals were available.

Genotyping of rs2453533-*GATM* was performed by polymerase chain reaction (PCR) followed by restriction fragment length polymorphism (RFLP). We selected the primers and restriction enzymes using the freely available software GeneRunner (Hastings Software Inc.). The PCR conditions were as follows: initial denaturation 95 °C for 10 min followed by 35 cycles of 95 °C for 30 s, 55 °C for 30 s and 72 °C for 30 s. PCR products were incubated with the restriction enzyme ApoI (Thermo Scientific, Waltham, MA, USA ) at 37 °C overnight. Digested products were resolved in 2% agarose gel.

Genotyping of rs4293393-*UMOD/PDILT*, rs1047891-*CPS1* and rs989566-*BCAS3* was performed using PCR followed by high-resolution melting (HRM), using the LightCycler 480 instrument for both steps (Roche Diagnostics, Indianapolis, IN, USA). Primers were designed using Primer3web (http://primer3.ut.ee/, accessed on 18 September 2016) and checked for potential primer dimer formation using OligoAnalyzer (http://eu.idtdna.com/calc/analyzer, accessed on 18 September 2016). We used 10-µL reaction volumes with 30 ng of DNA and LightCycler 480 High-Resolution Melting Master (Roche Diagnostics, Mannheim, Germany). The concentration of MgCl_2_ was optimized for each individual primer and was in the range of 2–3 mM. Cycling conditions were as follows: initial denaturation at 95 °C for 10 min, 40 cycles of denaturation (95 °C, 10s), annealing (58 °C, 15s) and extension (72 °C, 10 s). After amplification, the samples were heated to 95 °C for 1 min and rapidly cooled to 40 °C for 1 min at the rate of 1 °C/s, to induce heteroduplex formation before melting. Melting curve data were obtained from continuous fluorescence readings from 55 to 90 °C with a thermal transition rate of 0.02 °C/s. We based the genotype readings on negative first-derivate melting curves and comparison to reference samples (CEPH-Centre d’etude du polymorphisme humaine, Foundation Jean Dausset, Paris, France). We analyzed the data using the LightCycler 480 instrument software via two methods: gene scanning and Tm-calling. 

Genotypes for other selected SNPs were extracted from our genotype data bank. All genotypes were checked for Hardy-Weinberg equilibrium, and there were no deviations from it for any SNP in the control group. The reference alleles are presented as the effect allele defined by Wuttke et al. where the SNPs were identical, and as alleles in LD with the effect allele as defined by Wuttke et al. where the SNPs were not identical. All patient and control samples were processed together in the same workflows in the case of all respective genotyping methods to avoid batch effects.

### 2.2. Statistical Analysis

We reported numeric parameters as mean ± standard deviation (SD). We performed four case-control association analyses: all CKD patients versus controls, dialysis-independent patients versus controls, dialysis-dependent kidney failure patients versus controls and dialysis-dependent kidney failure versus dialysis-independent CKD patients. The case-control association analyses were performed using Fisher’s exact test. eGFRcrea and eGFRcys genetic association analysis was performed using multiple linear regression. Clinical parameter measurements were not available for control individuals; therefore, control individuals are excluded from this analysis. The statistical analysis was performed using SPSS software, version 26 (IBM Corp., Armonk, NY, USA). We used the Bonferroni correction for multiple testing with 12 comparisons; the corrected p (significance threshold, 0.05) was set to 0.004.

Using multiple linear regression, an α of 0.004 (Bonferroni correction for 12 SNPs–α = 0.05 / 12), a large effect size and 19 predictors, along with a sample size of at least 110 participants are needed to detect a significant association with a power of at least 80%.

Using Fisher’s exact test, an α of 0.004 (Bonferroni correction for 12 SNPs–α = 0.05 / 12), an odds ratio of approximately three or difference in proportions of approximately 0.25, a sample size of at least 70 cases and at least 200 controls are needed to detect a significant association with a power of at least 80% [18].

## 3. Results

### 3.1. Cohort Characteristics

There were no statistically significant differences in the distributions of age, sex, body mass index (BMI), systolic blood pressure, comorbidities or extent of edema between the dialysis-independent CKD and dialysis-dependent kidney failure groups. The dialysis-dependent kidney failure patients had higher albuminuria levels (*p* = 1.0 × 10^−6^). The 309 control individuals had a mean age of 42 years with an SD of 14.55 years, and there were 40% males and 60% females in the control group. The controls, like the patients, were of Slovenian origin. All control individuals were disease-free at the time of recruitment in the study. No other demographic information was available for the control individuals. The patient demographics and clinical phenotypes are shown in Table 1. 

### 3.2. Case-Control Association Analysis

We found a higher frequency of allele A of SNP rs2453533-*GATM* in dialysis-independent (non-dialysis) CKD patients (41%) compared to healthy controls (33%, corrected *p* = 0.020). Interestingly, the frequency of allele A was not significantly increased in dialysis-dependent kidney failure patients requiring hemodialysis. Additionally, we found a lower frequency of allele C of SNP rs4293393-*UMOD/PDILT* in CKD patients (15%) than in healthy controls (23%, corrected *p* = 0.042). Other associations with unadjusted p values lower than 0.05 did not remain significant after Bonferroni correction for multiple testing. We found no statistically significant differences between the genotypes of CKD patients and controls for the other investigated SNPs. Associations with adjusted *p* values < 0.05 are presented in Table 2. The complete results of this analysis are available in (Supplementary Table S2).

### 3.3. Genotype/Phenotype Analysis: Multiple Linear Regression Tests of Clinical Phenotypes

We tested the average eGFRcrea and eGFRcys against all dialysis-independent CKD patient genotypes. The eGFRcrea and eGFRcys were lower in carriers of the T allele of rs9895661*-BCAS3*. The results are presented in Table 3.

## 4. Discussion

In the present study, we demonstrate for the first time that the risk allele A of rs2453533-*GATM* is significantly more frequent in dialysis-independent CKD but not dialysis-dependent kidney failure patients. Böger et al. [12] previously identified loci that are significant in dialysis-independent CKD but not dialysis-dependent kidney failure patients, which could suggest differences in genetic architecture between dialysis-dependent kidney failure and dialysis-independent CKD patients. Surprisingly, few association studies have compared dialysis-independent CKD to dialysis-dependent kidney failure patients to replicate this observation. In our study, we provide further evidence for genetic differences between dialysis-independent CKD and dialysis-dependent kidney failure patients, and add the glycine amidinotransferase (*GATM*) gene locus to the loci associated with dialysis-independent CKD but not dialysis-dependent kidney failure patients. It is not unusual for an association to be present in dialysis-independent CKD, but not dialysis-dependent kidney failure patients. In a previous larger study, 11 of the 16 investigated eGFR-associated loci were also associated with CKD initiation risk, but only two with dialysis-dependent kidney failure. Differences in the genetic architectures of CKD and dialysis-dependent kidney failure resulting in differential mechanisms contributing to kidney disease initiation, incidence and progression, as well as premature cardiovascular mortality in CKD patients prior to dialysis-dependent kidney failure onset and the impact of non-genetic factors, have been suggested as possible explanations by Böger et al. [12]. General population eGFRcrea-associated SNPs have previously been linked to incident CKD in case-controlled association studies [12,16,17,19]. In most CKD GWAs, the “CKD” phenotype is defined as individuals in the general population with an eGFRcrea under 60 ml/min per 1.73 m^2^, and there is often no comparison between dialysis-independent CKD and dialysis-dependent kidney failure patients, measures of kidney disease progression, biopsy results or other markers of kidney pathology. Genetic association studies comparing dialysis-independent CKD and dialysis-dependent kidney failure as separate groups are still rare [12,20,21], even if dialysis-dependent kidney failure represents a widely used clinical CKD endpoint. In our study, the frequency of allele A of rs2453533-*GATM* was increased in the dialysis-independent CKD group compared to the healthy control group; however, the frequency of the allele was not significantly increased in a comparable dialysis-dependent kidney failure group. Allele A of rs2453533-*GATM* has been linked to decreased eGFRcrea (increased serum creatinine) on a population level, but no effect on eGFRcys has been identified [16,22]. According to Köttgen et al. this suggests that it is primarily a locus which affects creatinine production rather than a kidney function [16]. Our study has also not identified any associations of rs2453533-*GATM* with CKD-related clinical phenotypes. As eGFRcrea measurement is a principal part of the CKD diagnostic workup, patients with genetically increased serum creatinine levels could be diagnosed with CKD as false positives. In this case, we would not expect carriers of A of rs2453533-*GATM* to progress to dialysis-dependent kidney failure since they have no significant underlying kidney disease, and this would explain the lack of association in the dialysis-dependent kidney failure group. The influence of allele A of rs2453533-*GATM* on serum creatinine levels requires further investigation. *GATM* encodes a mitochondrial enzyme that belongs to the amidinotransferase family and is involved in creatine biosynthesis, whereby it catalyzes the transfer of a guanidino group from L-arginine to glycine, resulting in guanidinoacetic acid, the immediate precursor of creatine. Since rs2453533 is located in the non-coding region near the *GATM* gene, it most likely influences *GATM* gene expression, resulting in higher creatine and consequently creatinine production rates. Indeed, the increased expression of *GATM* in skeletal muscle was associated with the A allele of rs2453533 in an eQTL analysis [23]. Unfortunately, no eQTL analysis for allele A of rs2453533 has been reported for the most relevant tissues (kidney, liver, pancreas) where *GATM* is most highly expressed and most of the body’s creatine is produced. Our study warrants such studies. In addition, the traditional notion that creatine is almost exclusively produced in major organs such as the kidney, liver and pancreas, and then utilized in muscle, has been challenged most recently, suggesting that skeletal muscle, and some other tissues where *GATM* is significantly expressed, could have their own active creatine-producing machinery [24].

However, we are not able to completely exclude the possibility that allele A of rs2453533-*GATM* has a role in CKD pathology. After all, our results associate this long-known eGFRcrea-associated locus with clinically manifest CKD. *GATM* is highly expressed in the kidney tubule and mutation in the *GATM* gene causes a recessive disorder of creatine deficiency and the autosomal dominant Renal Fanconi syndrome, which includes kidney failure [25]. Fully penetrant heterozygous missense mutations in *GATM* cause fibrillary deposition of *GATM* inside the mitochondria and lead to elongated, abnormal and dysfunctional mitochondria [25]. Kidney biopsies of affected patients have shown fibrosis and enlarged proximal tubule mitochondria with pathological *GATM* aggregates. Overexpression of mutant *GATM* and subsequent aggregation in mitochondria leads to oxidative damage, inflammation, fibrosis and increased cell death [26]. On the other hand, *GATM* knockout mice were viable and no aminoaciduria or glucosuria was observed, indicating that a lack of *GATM* did not directly affect the function of proximal tubules. Rather, Reichold et al. suggest that an excess of mutant *GATM* proteins in the mitochondria triggers a pathologic cascade that eventually leads to the signs and symptoms in the patients with Fanconi syndrome due to a chronic inflammatory response as a reaction to impaired mitochondrial degradation [25]. Creatine, which enables the recycling of ATP molecules, is primarily synthesized in the kidneys and the liver, and primarily stored in skeletal muscle [27]. The turnover of creatine to creatinine is about 2g daily in a 70 kg male [28]. The normal expression of *GATM*, the first enzyme in creatine biosynthesis, is higher in the kidney (specifically in the proximal tubular cells (PTCs)) than in most other human tissues [29]. PTCs have one of the largest mitochondrial compartments because their transport activity requires great amounts of energy [30]. Evidence about the effect of rs2453533 on *GATM* or involvement in kidney dysfunction is lacking, and to our knowledge, this locus was not previously replicated in any case-control study of clinically diagnosed CKD patients compared to healthy controls. To summarize, *GATM* has a known role in CKD pathology in the case of a known missense gain-of-function mutation, while loss of function in knockout mouse experiments shows no influence on kidney function. It is likely that in contrast with missense mutations causing *GATM* structural changes, mutations causing slight changes in *GATM* expression have no influence on kidney function. However, the functional role of allele A of rs2453533-*GATM* on kidney pathology and its relationship to creatine and creatinine production still require further investigation.

Our case-control analysis also identified an association between a lower frequency of allele C of SNP rs4293393-*UMOD/PDILT* and CKD. The association was significant after adjustment only in the combined group of all CKD patients. This is consistent with Böger et al. [12], who identified the association of LD SNP rs12917707 with incident CKD, but not dialysis-dependent kidney failure. Of note, rs4293393-*UMOD/PDILT* and rs11864909-*UMOD/PDILT* are located relatively close to each other (~326 kbp), but outside of reliable LD. In both cases, the frequency of the minor allele is lower in affected individuals. Our findings are consistent with previous studies of the better-known rs4293393-*UMOD/PDILT*, the minor allele C, which decreases the odds of developing CKD [16]. In our study, rs4293393-*UMOD/PDILT* correlates with CKD. A previous much larger study by Böger et al. has described CKD risk variants that correlate with dialysis-independent CKD but not dialysis-dependent kidney failure, including in *UMOD* [12]. According to Gorski et al. the minor T allele of the SNP rs12917707-*UMOD* was associated with a reduced risk of incident CKD [12] and was later shown to affect eGFR decline [31]. In the CKDGen consortium, the same allele was associated with higher eGFR [17]. 

Köttgen et al. [17] and Okada et al. [32] have previously identified the association between allele C of rs9895661-*BCAS3* and decreased eGFRcrea. However, several newer population-based studies, including Wuttke et al. [11] and another large recent study Hellwege et al. [33] describe T as the risk allele for eGFRcrea. In the CKDGen consortium, rs9895661 was classified under creatinine production loci [17]. In our study, both eGFRcrea and eGFRcys were decreased in CKD patients who were carriers of allele T. Thus we provide replication of this SNP’s effects in a patient-based cohort and contribute an additional CKD-related parameter (eGFRcys) associated with this risk allele.

The advantages of our study were good patient group comparability (no significant differences between the dialysis-independent CKD and dialysis-dependent kidney failure groups in age, sex, BMI, systolic blood pressure, presence of comorbidities or extent of edema), availability of clinical parameters and relatively long retrospective observation periods. Sample size represents a major limitation of our study. This is especially true for the dialysis-dependent kidney failure group, which was the smallest (n = 73). In addition, clinical parameter measurements were only available for patients, and in the case of eGFRs, only for dialysis-independent CKD patients (and not controls or dialysis-dependent kidney failure patients). The dialysis-dependent kidney failure and dialysis-independent CKD patient groups were matched by age; however, the control group individuals were on average younger than the patients (see Results section). Assuming about a 10% lifetime risk of CKD incidence in the general population (mainly in later life), this is also a limitation of our case/control analysis. However, since genotypes generally do not change throughout life, and we can assume that the majority of our control group will not develop CKD in the coming few years, we have considered this an adequate approximation. A limitation of our analysis when studying the correlations between polymorphisms in/near the analyzed genes with eGFR in the group of dialysis-independent CKD patients (Table 3) is that we could not include a control group in this analysis, as we have no eGFR data available for controls. To compensate for a lack of a control group-based correction of eGFR values, we used the normalization of eGFR values by averaging several eGFR measurements over time in each patient, as well as age adjustment of these values in the linear regression. Following the great recent successes in mapping eGFR loci in the general population, we encourage researchers to further focus on phenotypes related to CKD progression and pathology using parameters beyond serum creatinine measurements. We also advise clinicians to interpret serum creatinine measurements with caution and with the knowledge that certain genetic markers might correlate with increased serum creatinine values in the absence of kidney disease. 

## 5. Conclusions

We independently replicated GWAs’ results for rs2453533-*GATM* in a Slovenian clinically diagnosed CKD patient cohort and demonstrated for the first time that the frequency of the risk A allele of rs2453533*-GATM* is increased in dialysis-independent CKD but not in dialysis-dependent kidney failure patients. Because *GATM* is an enzyme in the creatinine production chain, and it could only be associated with eGFRcrea but not eGFRcys in population-based GWAs, it was previously assumed that its polymorphisms likely have no influence on kidney function. Our results could be interpreted as showing a higher likelihood of individuals with genetically increased serum creatinine levels but no significant underlying kidney disease being diagnosed with CKD as false positives. The association was notably absent in a comparable dialysis-dependent kidney failure group. This would mean that physicians might benefit from having access to genetic information on these individuals when interpreting serum creatinine measurements. To avoid false-positive CKD diagnoses, eGFRcrea should be interpreted with caution, and other diagnostic criteria including eGFRcys should be preferred in the carriers of creatinine-increasing variants. However, our replication of this SNP in a case-control study between a clinically diagnosed CKD patient group and healthy controls might warrant further study of this polymorphism’s influence on kidney function, and the investigation of clinical features and structural changes on larger patient samples. A decrease in *GATM* showed no effect in knockout mouse studies, while a known *GATM* missense mutation causes excessively expressed mutant *GATM* proteins to deposit in mitochondria and interfere with their function. Interpreting general population-based eGFRcrea associations in relation to CKD pathology can often prove challenging. By focusing on clinical phenotypes related to disease manifestation and progression rather than just eGFR variation in the healthy population, researchers in the field of CKD genetics should be able to discover markers with stronger connections to CKD pathology. In the meantime, clinicians should keep in mind that certain polymorphisms may affect the levels of serum creatinine in the absence of kidney disease, and interpret eGFRcrea results with caution.

## Figures and Tables

**Table 1:** Demographics and clinical phenotypes in the examined patient groups.

Characteristics	Dialysis-Independent CKD			Dialysis-Dependent Kidney Failure			*p*
	n	mean/%	SD	n	mean/%	SD	
Age (in years)	135	69.9	15.3	73	68.5	14.0	0.517 *
Sex (male/female) %	135	45 / 55		73	59/41		0.058 #
BMI (kg/m ^2^)	42	28.4	5.2	53	27.7	5.9	0.547 *
eGFRcrea (ml/min/1.73 m^2^)	135	30.3	16.7	/	/	/	
eGFRcys (ml/min/1.73 m^2^)	135	30.9	19.7	/	/	/	
Albuminuria (0–4)	135	0.8	0.9	73	1.7	0.7	<0.001 *
Systolic blood pressure (mmHg)	135	152.4	17.4	73	154.0	13.5	0.460 *
N. of AH, DM, CVD	135	1.6	0.7	73	1.6	0.8	1.000 *
Edema	135	0.9	0.9	73	0.8	0.9	0.445 *
Arterial hypertension (AH) %	135	88 **	/	73	85 **	/	0.523 #
Diabetes mellitus (DM) %	135	36 **	/	73	32 **	/	0.520 #
Cardiovascular disease (CVD) %	135	39 **	/	73	38 **	/	1.000 #

N, the number of individuals with non-missing measurements; mean, the mean value in each individual’s retrospective observation period; SD, standard deviation; * *t*-test for independent samples; ** count; # Fisher’s exact test. Significant Bonferroni adjusted *p*-values are shown in bold.

**Table 2:** Associations in Fisher’s exact case-control analysis between CKD patient groups and the healthy control group with unadjusted p values below 0.05.

Test	SNP	Gene	EA	f(p)	f(c)	*p*	*p*(B)
All CKD patients vs. controls	rs1047891	*CPS1*	A	0.33	0.25	0.012	0.143
	rs2453533	*GATM*	C	0.59	0.67	0.013	0.154
	rs1145084	*GATM*	G	0.59	0.67	0.037	0.448
	rs4293393	*UMOD/PDILT*	C	0.15	0.23	0.004	**0.042**
	rs11864909	*UMOD/PDILT*	C	0.75	0.65	0.008	0.091
CKD dialysis-independent vs. controls	rs1047891	*CPS1*	A	0.35	0.25	0.007	0.078
	rs2453533	*GATM*	C	0.56	0.67	0.002	**0.020**
	rs1145084	*GATM*	G	0.57	0.67	0.006	0.076
	rs4293393	*UMOD/PDILT*	C	0.15	0.23	0.009	0.108
Kidney failure vs. controls	rs11864909	*UMOD/PDILT*	C	0.78	0.65	0.006	0.067
Kidney failure (f(p)) vs. CKD dialysis-independent (f(c))	rs1145084	*GATM*	G	0.65	0.55	0.045	0.416

EA, effect allele; f(p), frequency of the effect allele in patients; f(c), frequency of the effect allele in controls; *p*, Fisher Exact *p*-value; *p*(B), Bonferroni adjusted Fisher Exact *p*-value; kidney failure, dialysis-dependent kidney failure. Significant Bonferroni adjusted p-values are shown in bold.

**Table 3:** Associations with average eGFRcys and eGFRcrea in the dialysis-independent group.

Variable		Linear Regression Result				
eGFRcys		B	SE	β	*p*	*p*(B)
Sex (female vs male)		−4.45	2.28	−0.11	0.052	ns
Age (in years)		−0.28	0.08	−0.21	<0.001	**0.008**
Arterial hypertension		−7.26	3.65	−0.12	0.048	ns
Diabetes mellitus		0.71	2.64	0.02	0.788	ns
Cardiovascular disease		−2.04	2.50	−0.05	0.414	ns
Edema		−2.61	1.22	−0.13	0.033	ns
Systolic blood pressure		0.02	0.07	0.02	0.730	ns
rs1047891	A vs C	−1.00	2.57	−0.02	0.697	ns
rs11746443	A vs G	−2.89	2.58	−0.07	0.264	ns
rs2279463	C vs T	−7.23	4.12	−0.10	0.081	ns
rs10275044	T vs A	−3.30	3.21	−0.06	0.304	ns
rs7805747	G vs A	−6.68	2.56	−0.16	0.010	ns
rs685270	C vs T	−6.06	2.45	−0.15	0.014	ns
rs2453533	C vs A	−0.96	13.0	−0.02	0.941	ns
rs1145084	G vs A	1.94	12.97	0.05	0.881	ns
rs4293393	C vs T	−1.21	3.38	−0.02	0.720	ns
rs11864909	C vs T	−3.60	2.66	−0.08	0.178	ns
rs9895661	T vs C	11.05	2.79	−0.24	<0.001	**0.001**
rs8091180	A vs G	−0.14	2.52	0.00	0.955	ns
eGFRcrea						
Sex (female vs male)		−3.01	1.98	−0.09	0.129	ns
Age (in years)		−0.23	0.07	−0.21	0.001	**0.016**
Arterial hypertension		−4.34	3.16	−0.08	0.171	ns
Diabetes mellitus		2.73	2.29	0.07	0.233	ns
Cardiovascular disease		−3.58	2.16	−0.10	0.099	ns
Edema		−1.93	1.05	−0.11	0.069	ns
Systolic blood pressure		0.02	0.06	0.02	0.754	ns
rs1047891	A vs C	−2.47	2.23	−0.07	0.269	ns
rs11746443	A vs G	−1.16	2.23	−0.03	0.605	ns
rs2279463	C vs T	−6.07	3.57	−0.10	0.090	ns
rs10275044	T vs A	−3.01	2.78	−0.07	0.280	ns
rs7805747	G vs A	−4.01	2.22	−0.11	0.072	ns
rs685270	C vs T	−3.88	2.12	−0.11	0.069	ns
rs2453533	C vs A	−0.59	11.26	−0.02	0.958	ns
rs1145084	G vs A	0.36	11.23	0.01	0.974	ns
rs4293393	C vs T	−2.07	2.93	−0.04	0.480	ns
rs11864909	C vs T	−3.98	2.30	−0.11	0.085	ns
rs9895661	T vs C	−7.83	2.41	−0.20	0.001	**0.017**
rs8091180	A vs G	2.06	2.19	0.06	0.347	ns

B: unstandardized coefficient, SE: standard error, β: standardized coefficient, *p*: unadjusted *p*-value, *p*(B): Bonferroni adjusted *p*-value. Significant Bonferroni adjusted *p*-values are shown in bold.

## Data Availability

The data presented in this study are available on request from the corresponding author. The data are not publicly available due to patient privacy reasons.

## Supplementary Materials

The following are available online at https://www.mdpi.com/article/10.3390/genes12060834/s1: Table S1, The selection of 12 SNPs in 10 significant loci from the study by Wuttke et al. and the linkage disequilibrium of our respective analyzed SNPs; Table S2, Case-control Fisher exact tests for all SNPs: Patient groups vs. healthy controls.

## References

[1] Couser W.G., Remuzzi G., Mendis S., Tonelli M. (2011). The contribution of chronic kidney disease to the global burden of major noncommunicable diseases. Kidney Int..

[2] Webster A.C., Nagler E.V., Morton R.L., Masson P. (2017). Chronic Kidney Disease. Lancet.

[3] Stel V.S., Brück K., Fraser S., Zoccali C., Massy Z.A., Jager K.J. (2017). International differences in chronic kidney disease prevalence: A key public health and epidemiologic research issue. Nephrol. Dial. Transplant..

[4] Bevc S., Hojs N., Knehtl M., Ekart R., Hojs R. (2019). Cystatin C as a predictor of mortality in elderly patients with chronic kidney disease. Aging Male.

[5] Tonelli M., Wiebe N., Guthrie B., James M.T., Quan H., Fortin M., Klarenbach S.W., Sargious P., Straus S., Lewanczuk R. (2015). Comorbidity as a driver of adverse outcomes in people with chronic kidney disease. Kidney Int..

[6] Tsai W.-C., Wu H.-Y., Peng Y.-S., Ko M.-J., Wu M.-S., Hung K.-Y., Wu K.-D., Chu T.-S., Chien K.-L. (2016). Risk Factors for Development and Progression of Chronic Kidney Disease: A Systematic Review and Exploratory Meta-Analysis. Medicine.

[7] Zhong J., Yang H.-C., Fogo A.B. (2017). A perspective on chronic kidney disease progression. Am. J. Physiol. Physiol..

[8] Heerspink H.J.L., Greene T., Tighiouart H., Gansevoort R.T., Coresh J., Simon A.L., Chan T.M., Hou F.F., Lewis J.B., Locatelli F. (2019). Change in albuminuria as a surrogate endpoint for progression of kidney disease: A meta-analysis of treatment effects in randomised clinical trials. Lancet Diabetes Endocrinol..

[9] Xu X., Eales J.M., Akbarov A., Guo H., Becker L., Talavera D., Ashraf F., Nawaz J., Pramanik S., Bowes J. (2018). Molecular insights into genome-wide association studies of chronic kidney disease-defining traits. Nat. Commun..

[10] Franceschini N., Le T., Akbarov A., Tomaszewski M., Morris A.P. (2018). Large-scale trans-ethnic genome-wide association study reveals novel loci, causal molecular mechanisms and effector genes for kidney function. Genet. Epidemiol..

[11] Wuttke M., Li Y., Li M., Sieber K.B., Feitosa M.F., Gorski M., Tin A., Wang L., Chu A.Y., Hoppmann A. (2019). A catalog of genetic loci associated with kidney function from analyses of a million individuals. Nat. Genet..

[12] Böger C.A., Gorski M., Li M., Hoffmann M.M., Huang C., Yang Q., Teumer A., Krane V., O’Seaghdha C.M., Kutalik Z. (2011). Association of eGFR-Related Loci Identified by GWAS with Incident CKD and ESRD. PLoS Genet..

[13] (2002). National Kidney Foundation K/DOQI clinical practice guidelines for chronic kidney disease: Evaluation, classification, and stratification. Am. J. Kidney Dis..

[14] Reference Range and Method Comparison Studies for Enzymatic and Jaffé Creatinine Assays in Plasma and Serum and Early Morning Urine—PubMed. https://pubmed.ncbi.nlm.nih.gov/10745982/.

[15] Machiela M.J., Chanock S.J. (2015). LDlink: A web-based application for exploring population-specific haplotype structure and linking correlated alleles of possible functional variants: Figure 1. Bioinformatics.

[16] Köttgen A., Glazer N.L., Dehghan A., Hwang S.-J.J., Katz R., Li M., Yang Q., Gudnason V., Launer L.J., Harris T.B. (2009). Multiple loci associated with indices of renal function and chronic kidney disease. Nat. Genet..

[17] Köttgen A., Pattaro C., Böger C. a, Fuchsberger C., Olden M., Glazer N.L., Parsa A., Gao X., Yang Q., Smith A. V (2010). New loci associated with kidney function and chronic kidney disease. Nat. Genet..

[18] Erdfelder E., FAul F., Buchner A., Lang A.G. (2009). Statistical power analyses using G*Power 3.1: Tests for correlation and regression analyses. Behav. Res. Methods.

[19] Wuttke M., Köttgen A. (2016). Insights into kidney diseases from genome-wide association studies. Nat. Rev. Nephrol..

[20] Zsom M., Fülöp T., Zsom L., Baráth A., Maróti Z., ENDREFFY E.E., BARÁTH Á., Maróti Z., ENDREFFY E.E. (2011). Genetic polymorphisms and the risk of progressive renal failure in elderly Hungarian patients. Hemodial. Int..

[21] Jamison R.L., Shih M.-C., Humphries D.E., Guarino P.D., Kaufman J.S., Goldfarb D.S., Warren S.R., Gaziano J.M., Lavori P. (2009). Veterans Affairs Site Investigators Effect of the MTHFR C677T and A1298C polymorphisms on survival in patients with advanced CKD and ESRD: A prospective study. Am. J. Kidney Dis..

[22] Pattaro C., Teumer A., Gorski M., Chu A.Y., Li M.M.M., Mijatovic V., Garnaas M., Tin A., Sorice R., Li Y.Y.Y.Y. (2016). Genetic associations at 53 loci highlight cell types and biological pathways relevant for kidney function. Nat. Commun..

[23] dbGaP Study Accession Number Phs000424.v8.p2. https://www.ncbi.nlm.nih.gov/projects/gap/cgi-bin/study.cgi?study_id=phs000424.v8.p2.

[24] Ostojic S.M. (2021). Creatine synthesis in the skeletal muscle: The times they are a-changin’. Am. J. Physiol. Endocrinol. Metab..

[25] Reichold M., Klootwijk E.D., Reinders J., Otto E.A., Milani M., Broeker C., Laing C., Wiesner J., Devi S., Zhou W. (2018). Glycine Amidinotransferase (GATM), Renal Fanconi Syndrome, and Kidney Failure. J. Am. Soc. Nephrol..

[26] Carney E.F. (2018). GATM mutations cause mitochondrial abnormalities and kidney failure. Nat. Rev. Nephrol..

[27] Barcelos R.P.P., Stefanello S.T.T., Mauriz J.L.L., Gonzalez-Gallego J., Soares F.A.A.A.A. (2016). Creatine and the Liver: Metabolism and Possible Interactions. Mini Rev. Med. Chem..

[28] Balsom P.D., Söderlund K., Ekblom B. (1994). Creatine in Humans with Special Reference to Creatine Supplementation. Sport. Med..

[29] Gene: GATM—ENSG00000171766. https://bgee.org/?page=gene&gene_id=ENSG00000171766.

[30] Courtoy P.J., Henriet P. (2018). GATM Mutations Cause a Dominant Fibrillar Conformational Disease in Mitochondria—When Eternity Kills. J. Am. Soc. Nephrol..

[31] Gorski M., Tin A., Garnaas M., McMahon G.M., Chu A.Y., Tayo B.O., Pattaro C., Teumer A., Chasman D.I., Chalmers J. (2015). Genome-wide association study of kidney function decline in individuals of European descent. Kidney Int..

[32] Okada Y., Sim X., Go M.J., Wu J.-Y., Gu D., Takeuchi F., Takahashi A., Maeda S., Tsunoda T., Chen P. (2012). Meta-analysis identifies multiple loci associated with kidney function-related traits in east Asian populations. Nat. Genet..

[33] Hellwege J.N., Velez Edwards D.R., Giri A., Qiu C., Park J., Torstenson E.S., Keaton J.M., Wilson O.D., Robinson-Cohen C., Chung C.P. (2019). Mapping eGFR loci to the renal transcriptome and phenome in the VA Million Veteran Program. Nat. Commun..

